# Anatomical analysis of the posterior elbow joint capsule and small muscle bundle deep to the triceps brachii: a combined cadaveric and in vivo study

**DOI:** 10.1186/s12891-026-10214-3

**Published:** 2026-07-11

**Authors:** Rintaro Yamamoto, Akimoto Nimura, Hitomi Fujishiro, Satoru Muro, Keiichi Akita

**Affiliations:** 1 Department of Clinical Anatomy, Graduate School of Medical and Dental Science, Institute of Science Tokyo, Tokyo, Japan; 2 Research Infrastructure Management Center, Institute of Science Tokyo, Tokyo, Japan; 3 Department of Functional Joint Anatomy, Institute of Biomedical Engineering, Institute of Science Tokyo, 1-5-45 Yushima, Bunkyo-ku, Tokyo, 113-8519 Japan; 4 Department of Physiological and Anatomical Science, Graduate School of Medical and Dental Sciences, Institute of Science Tokyo, Tokyo, Japan

**Keywords:** Anatomy, Articular, Ultrasonography, Ultrasound, Innervation, Rehabilitation, Stiffness

## Abstract

**Background:**

Restricted elbow flexion sometimes persists even after stretching the triceps brachii. Although prior descriptions have noted that a small muscle bundle is located between the posterior elbow joint capsule and the triceps brachii, its anatomical relationship with surrounding muscles and dynamic changes during forearm rotation remain unclear. We hypothesized that the small muscle bundle separates from the triceps and contributes to forearm rotation. This study aimed to clarify the anatomical structure of the small muscle bundle and its dynamic changes during forearm rotation.

**Methods:**

We analyzed 19 elbow specimens from 16 Japanese cadavers, donated to the Department of Anatomy, Institute of Science Tokyo, to identify the small muscle bundle covering the joint capsule. One specimen was excluded because of severe osteophytes, leaving 18 specimens randomly allocated to three groups (10, 3, and 5 specimens in the macroscopic examination, intramuscular nerve distribution examination, and histological examination groups, respectively). Additionally, we investigated 10 elbows of five healthy volunteers to evaluate dynamic changes in the small muscle bundle and joint capsule using ultrasonography during forearm rotation.

**Results:**

Anatomically, deep to the triceps brachii, a small muscle bundle covering the posterior elbow joint capsule was identified. The small muscle bundle was continuous laterally with the anconeus muscle and was separated from the triceps by loose connective tissue. The small muscle bundle and the anconeus were innervated by the same branches of the radial nerve. Ultrasonically, the small muscle bundle was found to be significantly thicker during forearm supination (6.0 ± 1.4 mm) than pronation (4.1 ± 1.0 mm; *p* = 0.002), and the distance between the olecranon fossa and the posterior edge of the joint capsule was significantly shorter during supination (8.9 ± 1.1 mm) than pronation (9.6 ± 1.1 mm; *p* = 0.023).

**Conclusions:**

The small muscle bundle formed a muscular complex with the anconeus and was distinct from the triceps. The small muscle bundle deep to the triceps may act during forearm rotation, affecting the posterior elbow joint capsule. These anatomical and ultrasonographic findings may contribute to a better understanding of the pathophysiology of restricted elbow flexion.

## Background

The elbow joint is a compound joint that performs flexion–extension and pronation–supination. The range of motion of the elbow joint plays an important role in the functional use of the upper limbs during daily activities and sports performances. However, post-traumatic and postoperative elbow stiffness is common, often resulting in significant limitation of motion [1]. Adhesions between the triceps brachii and the posterior elbow joint capsule have been reported to contribute to restricted elbow flexion [2]. Although early rehabilitation has been considered effective for restricted elbow flexion [3, 4], stretching the triceps brachii, a standard rehabilitation exercise, has been described as sometimes not improving the range of motion [5, 6]. Accordingly, effective treatment strategies for restricted elbow flexion have not been established because there is a lack of anatomical knowledge regarding the posterior elbow joint capsule and muscles covering the joint capsule.

The attachment of the joint capsule and the relationship between the joint capsule and its surrounding muscles contribute to the joint stabilization mechanism and pathology in some joints [7–10]. The posterior part of the elbow joint is generally composed of the posterior capsule and the triceps. The common tendon of the triceps has been reported to insert into the distal part of the olecranon with a distinct bony impression [11]. The joint capsule attaches to the proximal part of the olecranon [12–15]. However, the precise attachment width of the joint capsule at the olecranon and the complementary attachment relationship with its surrounding muscles remain unclear.

Some reports have described a small muscle bundle deep to the triceps as either a part of the medial head of the triceps or an independent muscle bundle that covers the joint capsule. This small muscle bundle has been referred to as the “subanconeus muscle” or the “articularis cubiti muscle” [16–18]. However, the attachment, layer relationship with surrounding muscles, and independence of this small muscle bundle remain unclear. We hypothesized that a distinct muscle bundle, different from the triceps, exists between the posterior capsule and the triceps.

Additionally, the small muscle bundle covering the posterior capsule may be anatomically adjacent to the anconeus muscle, which is located at the posterolateral aspect of the elbow. The anconeus has been reported to be active during forearm supination [19, 20]. Given its anatomical proximity, the small muscle bundle may also contribute to forearm rotation. However, the anatomical relationship of the small muscle bundle with the anconeus remains unclear, and a purely anatomical study cannot clarify its function regarding forearm rotation. Recent anatomical studies in other joints have examined function by combining cadaver studies with ultrasonography [21, 22]. Ultrasound can visualize posterior elbow structures, including the triceps and joint capsule [23], and quantitatively evaluate their dynamic changes during forearm rotation. We also hypothesized that the small muscle bundle has a close relationship with the anconeus, and the thickness of the small muscle bundle dynamically changes and affects the joint capsule during forearm rotation.

In the present study, we aimed to clarify the small muscle bundle covering the posterior elbow joint capsule and reveal the olecranon attachment of the joint capsule using macroscopic, intramuscular nerve distribution, and histological examinations. In addition, we aimed to analyze the dynamic changes in the small muscle bundle and joint capsule during forearm rotation in vivo using ultrasonography. Insight into the structure and function of the muscle bundle covering the posterior capsule may provide clues for understanding the pathophysiology of restricted elbow flexion.

## Methods

### Preparation of elbow specimens

This study was approved by the Institutional Review Board of Institute of Science Tokyo (M2018-097). The study sample included 19 elbows (10 right and 9 left) obtained from 16 Japanese cadavers donated to the Department of Anatomy, Institute of Science Tokyo—eight male and eight female—with a mean (range) age at death of 82.8 (62–93) years. Elbow joints with evidence of a prior surgery or joint deformities were excluded. The entire process was conducted in accordance with the Japanese guidelines entitled “Ethical Guidelines for Medical and Health Research Involving Human Subjects.” Cadavers were fixed in 8% formalin and stored in 30% ethanol. The upper extremities were harvested as follows. The shoulder joint capsule was removed to separate the humeral head from the scapula, and a distal cut was made to preserve the proximal one-third of the forearm. The long head of the triceps brachii was separated from its scapular origin.

The osseous morphology of the elbow specimens was examined using micro-computed tomography (micro-CT; InspeXio SMX-100CT; Shimadzu Corp., Kyoto, Japan). The resolution of the images was 200 μm. We reconstructed three-dimensional (3D) images from a series of micro-CT images using ImageJ software (version 1.54; National Institutes of Health, Bethesda, MD, USA) to eliminate elbows with severe osteophytes and to confirm the sagittal section levels, rather than to perform osseous morphological analysis. One elbow specimen was excluded because severe osteophytes were identified in the 3D images. The remaining 18 elbow specimens were then randomly allocated to three groups: 10 in the macroscopic examination group, three in the intramuscular nerve distribution examination group, and five in the histological examination group.

### Macroscopic analysis of the joint capsule and pericapsular muscles at the posterior elbow joint

We used 10 specimens for the macroscopic analysis. The skin and subcutaneous fat were removed. We detached the triceps brachii from the olecranon from medial to lateral. Then, we identified a small muscle bundle deep in the triceps and detached it from the humerus and ulna from medial to lateral. Finally, we identified the joint capsule and detached it from the humerus and ulna in a medial-to-lateral direction.

The footprint of the joint capsule, the small muscle bundle, and the common tendon of the triceps brachii were measured after detachment from the olecranon. Attachment widths on the olecranon were measured at the olecranon tip and at the medial and lateral edges using a caliper. Measurements were performed by the same observer and repeated twice on separate days with randomized specimens. The between-day accuracy of footprint measurements was evaluated using the intraclass correlation coefficient (ICC). The reliability of footprint measurements was excellent, with a mean (range) ICC of 0.99 (0.985–0.993).

### Intramuscular nerve distribution of the triceps brachii, the small muscle bundle, and the anconeus

We used three specimens for intramuscular nerve distribution analysis. The skin and subcutaneous fat were removed, and the common tendon of the triceps brachii was detached. Then, we identified the branches of the radial nerve innervating the long, lateral, and medial heads of the triceps brachii, the small muscle bundle, and the anconeus. Finally, we dissected the course and extent of each nerve branch and investigated its intramuscular distribution under a stereomicroscope (magnification: 12×, Operation Microscope OLYMPUS OME-1000, Olympus Optical, Tokyo, Japan).

### Histological analysis

We used five specimens for histological analysis. After harvesting the elbow specimens, we stored the specimens at -80 °C with the elbow joint flexed at 90 degrees. We cut the frozen specimens into sagittal slices at the level of the olecranon tip and mid-level of the capitulum of the humerus (5 mm thick) using a diamond saw (ME-300, LUXO, Aichi, Japan). Each level of sectioning was identified by comparing the bony morphology of the section with the micro-CT images during specimen preparation. One specimen block containing the posterior elbow joint capsule and its surrounding muscles was obtained from each section. The specimen blocks were decalcified by soaking them for two weeks in Plank-Rychlo solution (AlCl3·6H2O [70.0 g/L], HCl [85.0 mL/L], and HCOOH [50.0 mL/L]) and were dehydrated [24]. The decalcified blocks were dehydrated and embedded in paraffin. We serially sliced the paraffin-embedded blocks into sections of 5-µm thickness at 500-µm intervals. Masson’s trichrome staining was used to stain the sections.

### In vivo ultrasonographic assessment of the posterior elbow joint

We investigated 10 elbows of five healthy volunteers (two men and three women) with a mean (range) age of 27.8 (23–35) years. Individuals with no history of elbow surgery were recruited. Ethical approval was obtained from the Institutional Review Board of Institute of Science Tokyo (I2025-121), and all procedures were performed in accordance with the Declaration of Helsinki (last revised in 2013) and the Japanese guidelines entitled “Ethical Guidelines for Medical and Health Research Involving Human Subjects.” All potential participants were informed about the study requirements, benefits, and risks; those desiring to participate provided written informed consent.

We used an ultrasound scanner (Aplio i900; Canon Medical Systems, Tokyo, Japan) with a 5–18 MHz linear transducer. The participants were seated with their elbows flexed at 90 degrees in the neutral forearm position and their hands on their hips. The examiner placed the transducer on the posterior elbow joint and maintained each participant’s forearm position during image acquisition. Sagittal ultrasonography was performed to visualize the muscle bundle covering the posterior elbow joint capsule at the level of the olecranon tip. While maintaining the elbow in a 90-degree flexed position, participants moved their forearms to maximum pronation and supination, each at 90 degrees. At each position, (1) the thickness of the small muscle bundle as an evaluation of its contraction and (2) the distance between the olecranon fossa and the posterior edge of the joint capsule as an evaluation of the effect on the capsule were measured using ImageJ along a line perpendicular to the fibrillar pattern of the small muscle bundle and passing through the deepest point of the olecranon fossa. One evaluator randomly repeated the measurements of the thickness of the small muscle bundle and the distance between the olecranon fossa and the posterior edge of the joint capsule for all volunteers by ultrasonography on two separate days, and the between-day reliability of the measurement was evaluated by calculating the ICC. The reliability of measurements of the thickness of the small muscle bundle and capsular distance were excellent, with a mean (range) ICC of 0.997 (0.992–0.999) and 0.981 (0.955–0.993), respectively.

### Statistical analysis

We used the statistical program EZR for Windows (version 1.68; Saitama Medical Center, Jichi Medical University, Saitama, Japan) [25]. Continuous data were reported as means and standard deviations. The average widths of the capsule attachments at the olecranon tip, lateral edge, and medial edge were compared using repeated-measures analysis of variance with Bonferroni correction for multiple comparisons. The average thickness of the muscle bundle and the average distance between the olecranon fossa and the posterior edge of the joint capsule at the maximum forearm pronation and supination were compared using a paired t-test. The significance level of the comparisons was set at *p* < 0.05. Measurement reliability was evaluated using the ICC, with an ICC value > 0.75 indicating excellent agreement.

## Results

### Macroscopic analysis of the joint capsule and pericapsular muscles at the posterior elbow joint

**Fig. 1:**
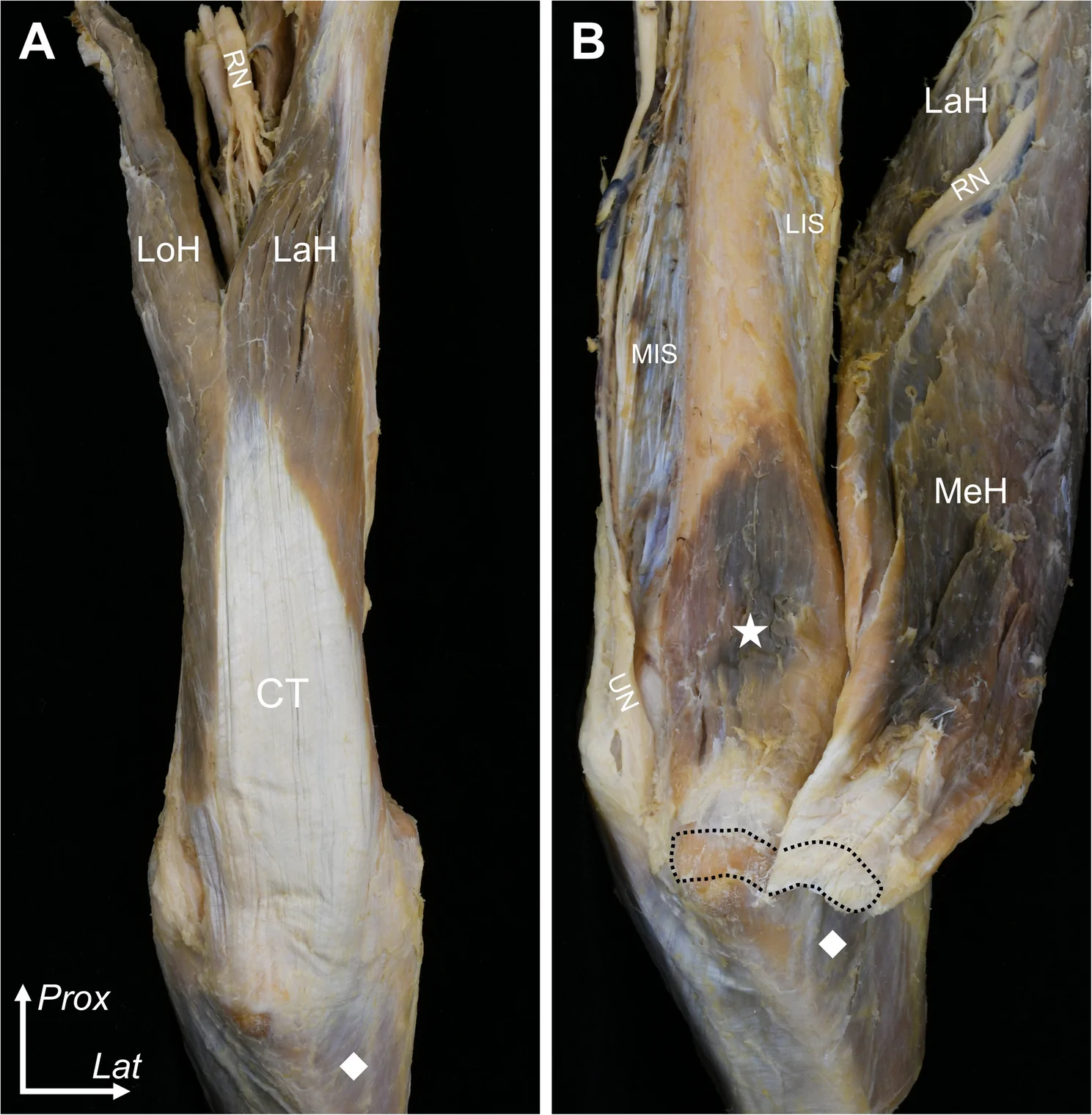
Positional relationship between the triceps brachii and small muscle bundle deep to the triceps brachii. **A** Posterior aspect of the right triceps brachii. **B** Detaching the triceps brachii from the humerus and the olecranon from medial to lateral reveals the small muscle bundle deep to the triceps brachii (*white star*). The black dotted area indicates the insertion site of the triceps brachii. The *white diamond* indicates the aponeurosis of the anconeus. CT, common tendon of triceps brachii; LoH, long head of triceps brachii; LaH, lateral head of triceps brachii; MeH, medial head of triceps brachii; LIS, lateral intermuscular septum; MIS, medial intermuscular septum; RN, radial nerve; UN, ulnar nerve; *Prox*, proximal; *Lat*, lateral

The triceps brachii was observed in the posterior aspect (Fig. 1A). After detaching the triceps brachii, the small muscle bundle that did not join the common tendon of the triceps brachii was identified deep to the medial head of the triceps brachii (white star in Fig. 1B).

**Fig. 2:**
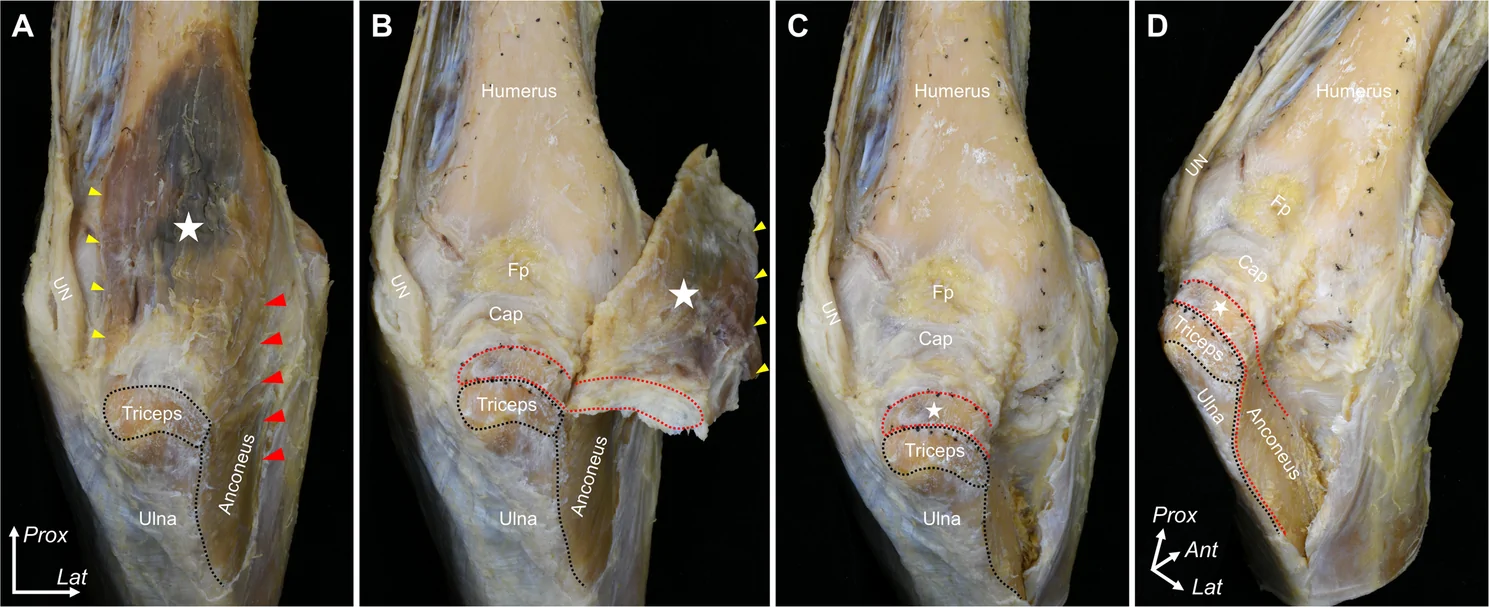
Macroscopic analysis of the small muscle bundle deep to the triceps brachii. **A** Posterior aspect of the right elbow after detaching the triceps brachii from the olecranon. The small muscle bundle (*white star*) laterally continued with the anconeus muscle (*red arrowheads*). **B** The small muscle bundle is detached from the olecranon from medial to lateral. The *yellow arrowheads* indicate the medial edge of the small muscle bundle. **C** Posterior aspect of the right elbow after detaching the small muscle bundle and the anconeus from the ulna. **D** Posterolateral aspect of the right elbow. The *black dotted area* indicates the insertion site of the triceps brachii. The *black dotted line* indicates the attachment of the aponeurosis of the anconeus. The *red dotted area* indicates the insertion site of the small muscle bundle. Cap, elbow joint capsule; Fp, fat pad; UN, ulnar nerve; *Prox*, proximal; *Lat*, lateral; *Ant*, anterior

The small muscle bundle laterally continued with the anconeus muscle (Fig. 2A). The small muscle bundle inserted into the olecranon proximal to the triceps via a thin, short aponeurosis (Fig. 2B and 2C). After removing the small muscle bundle and anconeus, the joint capsule and fat pad were exposed (Fig. 2C and D). From the posterolateral aspect, the border of the attachment area between the small muscle bundle and the anconeus was unclear (Fig. 2D). In all macroscopic specimens, the small muscle bundle deep to the triceps was identified, and its continuity with the anconeus was confirmed.

**Fig. 3:**
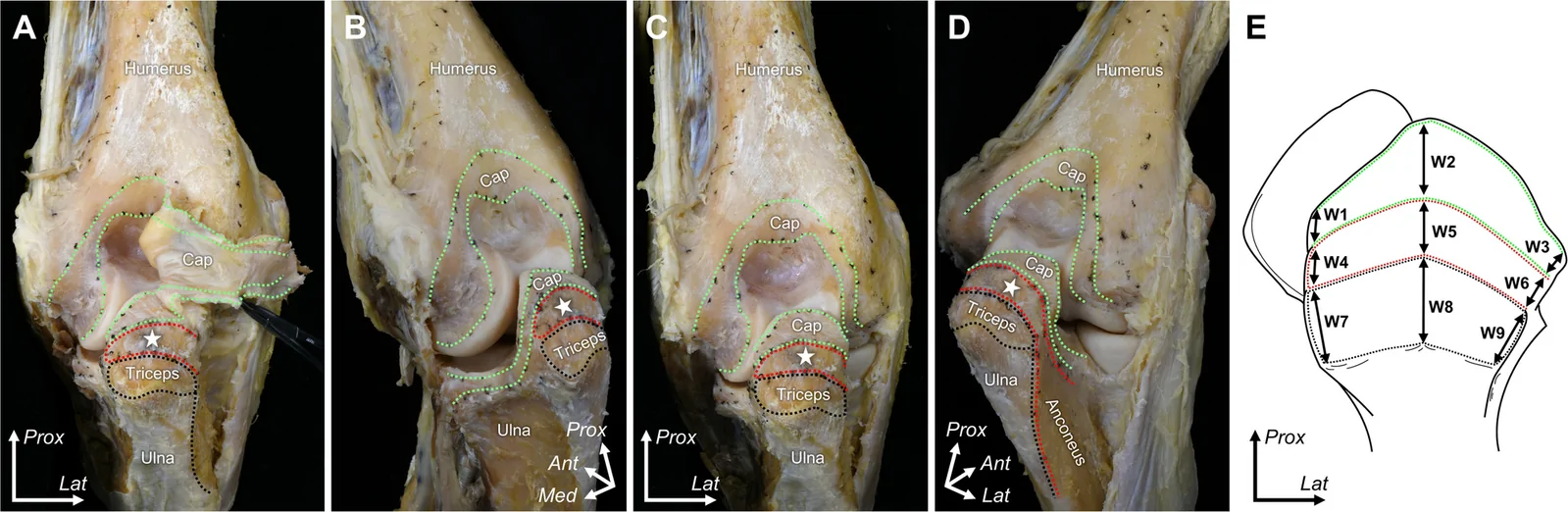
Attachment of the posterior elbow joint capsule at the olecranon. **A** Posterior aspect of the right elbow during detaching the elbow joint capsule (Cap) from the olecranon from medial to lateral. **B** Posteromedial aspect of the right elbow after detaching the Cap. **C** Posterior aspect of the right elbow. **D** posterolateral aspect of the right elbow. **E** Schematic diagram indicating the measurements of the attachment width on the right olecranon as shown in Table 1. The *black dotted area* indicates the insertion site of the triceps brachii. The *black dotted line* indicates the attachment of the aponeurosis of the anconeus. The *red dotted area* indicates the insertion site of the small muscle bundle deep to the triceps brachii (*white star*). The *green dotted area* indicates the capsular attachment. *Prox*, proximal; *Lat*, lateral; *Ant*, anterior; *Med*, medial

The posterior elbow joint capsule was attached to the olecranon proximal to the small muscle bundle (Fig. 3A–D). The widths of the capsular attachments to the olecranon significantly varied according to location (*p* < 0.001 for the medial edge of the olecranon (Fig. 3E, W1 = 2.5 ± 0.7 mm] vs. olecranon tip (Fig. 3E, W2 = 9.4 ± 1.6 mm], *p* < 0.001 for the olecranon tip (W2) vs. lateral edge of the olecranon (Fig. 3E, W3 = 2.6 ± 0.6 mm]). The width of the capsular attachment was relatively narrow at the medial edge of the olecranon, increased toward the olecranon tip, and decreased toward the lateral edge. The measurements of the attachment widths of the joint capsule, small muscle bundle, and common tendon of the triceps are shown in Fig. 3E and Table 1.

**Table 1:** Measurements of the attachment width on the olecranon

The location of the measurement	Average and standard deviation (mm)		
Joint capsule			
Medial edge (W1)	2.5	±	0.7
Olecranon tip (W2)	9.4	±	1.6
Lateral edge (W3)	2.6	±	0.6
Small muscle bundle			
Medial edge (W4)	3.4	±	0.6
Olecranon tip (W5)	7.6	±	0.9
Lateral edge (W6)	5.5	±	0.8
Common tendon of the triceps brachii			
Medial edge (W7)	7.0	±	0.9
Olecranon tip (W8)	9.6	±	1.5
Lateral edge (W9)	6.2	±	0.7

Locations of measurements are demonstrated in Fig. 3E

### Intramuscular nerve distribution of the triceps brachii, the small muscle bundle, and the anconeus

**Fig. 4:**
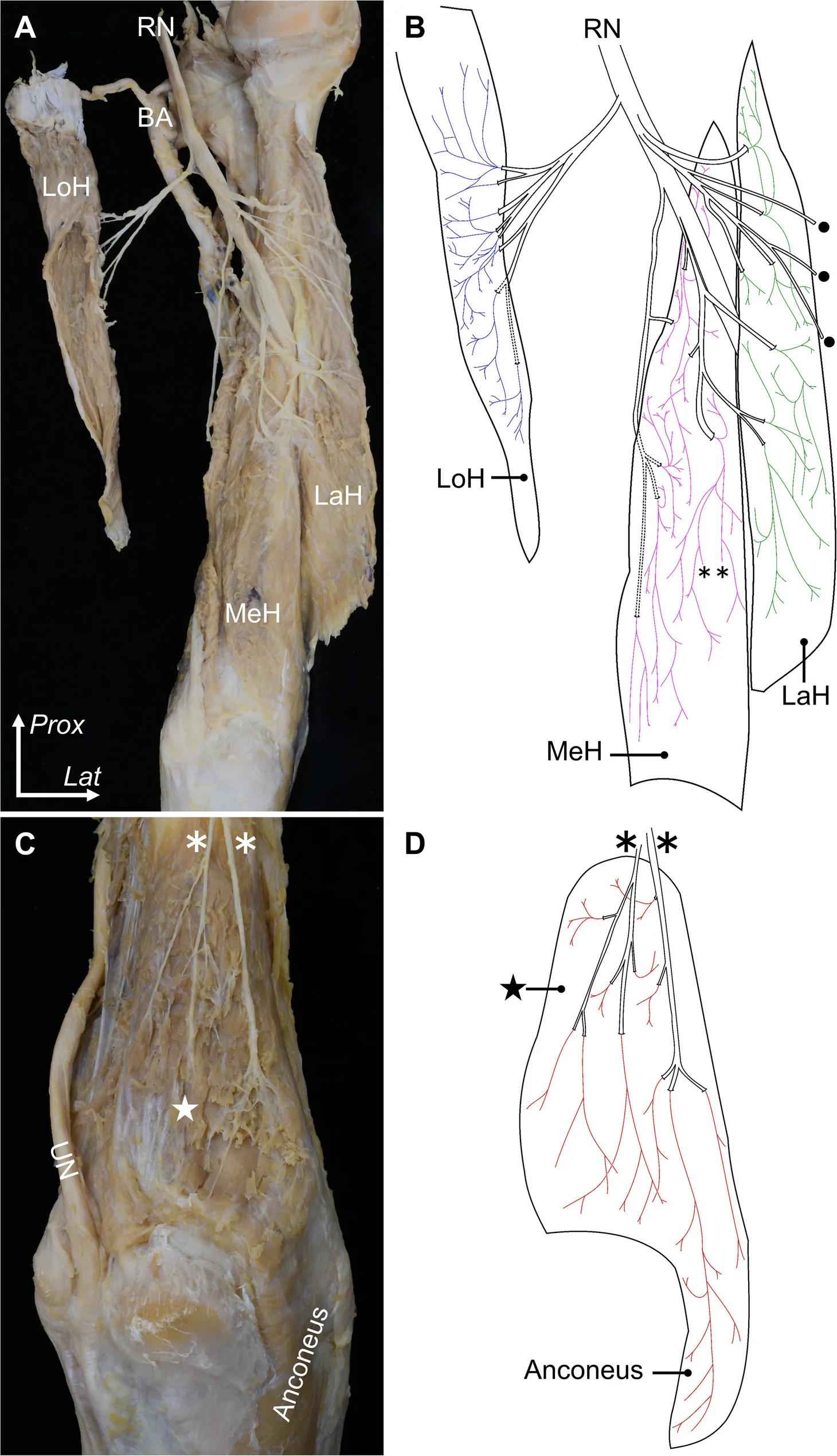
Intramuscular nerve distribution of the triceps brachii, the small muscle bundle, and the anconeus. **A** Posterior aspect of the right triceps brachii after removing the common tendon of the triceps brachii. The long head (LoH) is reflected medially. **B** Line drawing of the intramuscular nerve distribution of the triceps brachii corresponding to **A**. The *Blue*,* green*,* and pink lines* indicate nerve branches from the radial nerve (RN) within the LoH, lateral head (LaH), and medial head (MeH), respectively. The *black circles* indicate the branches extending toward the subcutaneous layer. The *asterisks* indicate the branches that penetrate the MeH. **C** After detaching the muscular portion of the triceps brachii from **A**. **D** Line drawing of the intramuscular nerve distribution of the small muscle bundle (*white star*) and the anconeus muscle. The branches (*asterisks*) innervate the small muscle bundle and the anconeus muscle (*orange lines*). BA, brachial artery; UN, ulnar nerve; *Prox*, proximal; *Lat*, lateral

The branch originating from the proximal part of the radial nerve innervated the long head of the triceps brachii. The subsequent branches originating from the radial nerve innervated the proximal portion of the lateral head of the triceps, with some branches passing subcutaneously. The subsequent branches originating from the radial nerve innervated the medial head and the distal portion of the lateral head of the triceps (Fig. 4A and 4B). Some branches innervating the lateral portion of the medial head penetrated the medial head and extended toward its deeper layer (asterisks in Fig. 4A and 4B). These branches ran between the medial head and the small muscle bundle and innervated the small muscle bundle. The lateral portion of these branches extended distally and innervated the anconeus muscle (Fig. 4C and 4D).

### Histological analysis of the posterior elbow joint

**Fig. 5:**
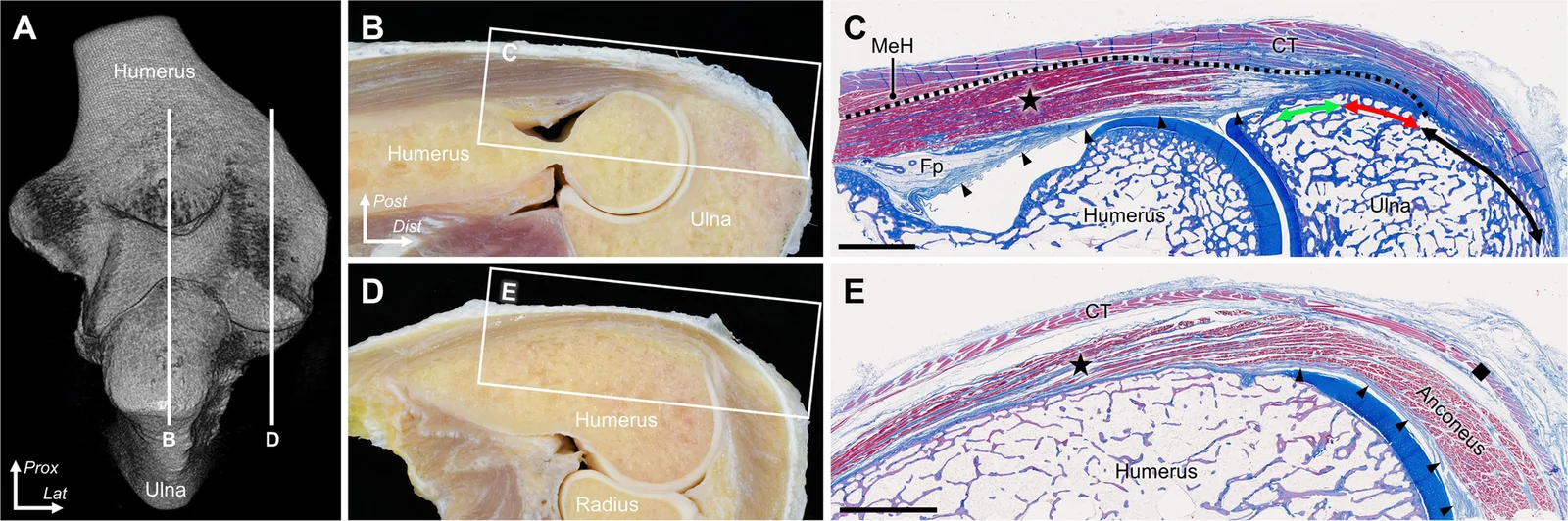
Histological analysis of the posterior elbow joint on sagittal sections (Masson’s trichrome staining). **A** Micro-computed tomography image of the posterior aspect of the right elbow in 90-degree flexion. **B** Sagittal section at the level of the olecranon tip (*white line B* in **A**). **C** Histological section of the boxed region in **B**. The *black dotted line* indicates loose connective tissue separating the small muscle bundle deep to the triceps brachii (*black star*) from the medial head (MeH) and the common tendon of the triceps brachii (CT) proximally. Distally, the boundary between the small muscle bundle and the CT was defined according to the bony prominence of the olecranon and the direction of these fibers. The *black double-headed arrow* indicates the attachment of the CT. The *red double-headed arrow* indicates the attachment of the small muscle bundle. The *green double-headed arrow* indicates the attachment of the joint capsule. **D** Sagittal section at the mid-level of the capitulum of humerus (*white line D* in **A**). **E** Histological section of the boxed region in **D**. The *black arrowheads* indicate the joint capsule. The *black diamond* indicates the aponeurosis of the anconeus. Fp, fat pad; Prox, proximal; Lat, lateral; Post, posterior; Dist, distal; scale bars, 5 mm

We performed a histological examination of the sagittal sections at the olecranon tip level and mid-level of the humeral capitulum (Fig. 5A). Located deep to the medial head and the common tendon of the triceps brachii, the small muscle bundle extended toward the olecranon (Fig. 5B and 5C). Proximally, the small muscle bundle was separated from the medial head and common tendon of the triceps brachii by loose connective tissue. Distally, at the olecranon insertion, the boundary between the small muscle bundle and the common tendon was defined according to the bony prominence of the olecranon and the direction of these fibers (black dotted line in Fig. 5C). The small muscle bundle attached to the olecranon between the joint capsule and the common tendon, closely covering the joint capsule and fat pad (Fig. 5C). Laterally, the small muscle bundle continued with the anconeus muscle, covering the joint capsule (Fig. 5D and 5E).

### Ultrasonographic imaging of the posterior elbow joint

**Fig. 6:**
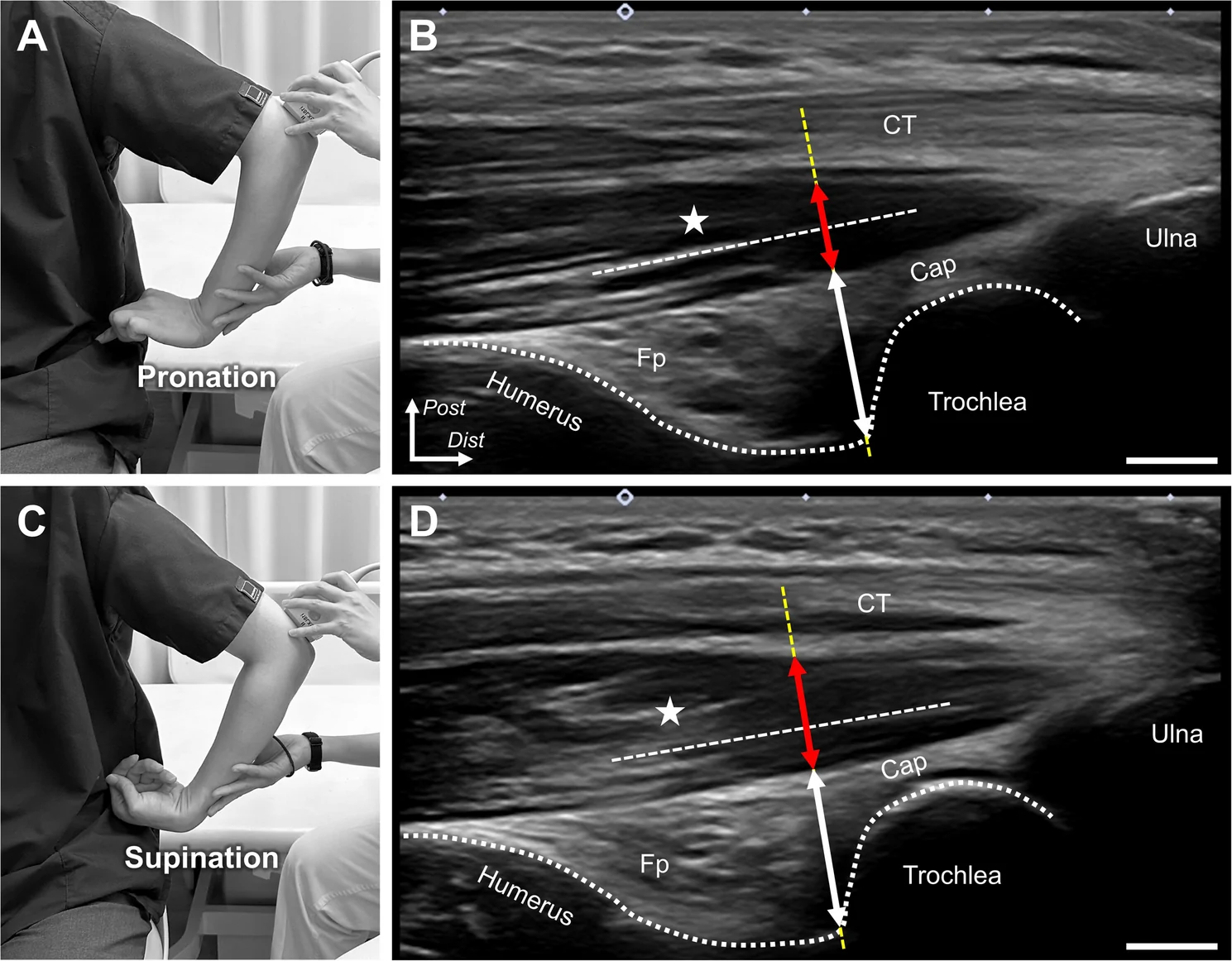
Ultrasonographic examination of the posterior elbow joint. **A**, **C** Elbow joint position and transducer placement for ultrasonographic examination. **B**, **D** Ultrasonographic images. The *white star* indicates the small muscle bundle between the common tendon of the triceps brachii (CT) and the joint capsule (Cap). The thickness of the small muscle bundle (*red double-headed arrows*) and the distance between the olecranon fossa and the posterior edge of the Cap (*white double-headed arrows*) were measured along a line passing through the deepest point of the olecranon fossa (*yellow dashed lines*), perpendicular to the fibrillar pattern of the muscle bundles (*white dashed lines*) as shown in Table 2. The *white dotted lines* indicate the edge of the humerus. **A** Ultrasonographic examination in the maximal forearm pronation with the elbow flexed at 90 degrees. **B** Sagittal view at the level of the olecranon tip in the maximal forearm pronation. **C** Ultrasonographic examination in the maximal forearm supination with the elbow flexed at 90 degrees. **D** Sagittal view at the level of the olecranon tip with the maximal forearm supination. Fp, fat pad; *Post*, posterior; *Dist*, distal; scale bars, 5 mm

At the level of the olecranon tip, a small muscle bundle was visualized between the common tendon of the triceps and the posterior elbow joint capsule. The thickness of the small muscle bundle during maximal forearm pronation was significantly less (4.1 ± 1.0 mm; Fig. 6A and 6B) than during maximal supination (6.0 ± 1.4 mm; *p* = 0.002; Fig. 6C and 6D). The distance between the olecranon fossa and the posterior edge of the joint capsule during maximal pronation was significantly greater (9.6 ± 1.1 mm; Fig. 6A and 6B) than during maximal supination (8.9 ± 1.1 mm; *p* = 0.023; Fig. 6C and 6D). All measurements are listed in Table 2.

**Table 2:** Measurements were performed using sagittal ultrasonography during forearm rotation at the olecranon tip level

The location of the measurement	Average and standard deviation (mm)						*P* value
	Maximum pronation			Maximum supination			
Thickness of the small muscle bundle	4.1	±	1.0	6.0	±	1.4	0.002
Distance between the olecranon fossa and the posterior edge of the joint capsule	9.6	±	1.1	8.9	±	1.1	0.023

Locations of measurements are demonstrated in Fig. 6

*P* value < 0.05 indicates a significant difference

## Discussion

In the present study, we found that the small muscle bundle located deep to the triceps brachii was continuous laterally with the anconeus muscle, closely covered the joint capsule and fat pad, was separated from the triceps by loose connective tissue, and attached directly to the olecranon. The nerve branches, running between the medial head and the small muscle bundle, innervated the small muscle bundle and the anconeus. In addition, the posterior elbow joint capsule was attached to the olecranon proximal to the attachment of the small muscle bundle, and the width of the capsular attachment was relatively wide at the olecranon tip and narrow at the medial and lateral edges of the olecranon. During forearm supination, the small muscle bundle thickened, and the joint capsule compressed toward the olecranon fossa, as observed in vivo using ultrasonography.

In the anatomical textbook, a gap is illustrated between the posterior elbow joint capsule and the triceps brachii at the olecranon [26]. Previous descriptions have identified a small muscle bundle between the joint capsule and the triceps as a part of the medial head, not as an independent muscle, referring to it as the “subanconeus muscle” or “articularis cubiti muscle” [16, 17]. In contrast, Benes et al. [18] described this bundle as an independent muscle distinct from the medial head. Moreover, the anconeus muscle has been described as a part of the medial head of the triceps brachii [27, 28]; however, the anatomical relationship between the anconeus and the small muscle bundle is unclear. Thus, whether the small muscle bundle is independent from the triceps and the anconeus remains contested in the literature. The current study revealed that the small muscle bundle covering the joint capsule was separated from the triceps by loose connective tissue and had an independent insertion at the olecranon. In addition, the small muscle bundle continued with the anconeus and was innervated by the same branches as the anconeus that penetrated the medial head. Based on these findings, the small muscle bundle could be interpreted as a part of the anconeus rather than the triceps brachii. Moreover, the anatomical textbook has mentioned that a part of the anconeus extended deep into the medial head [27]. The anatomical relationship between the small muscle bundle and the anconeus in the present study confirms this prior description, and the small muscle bundle could be considered a proximal extension of the anconeus.

In the anatomical textbook, the olecranon attachment of the posterior elbow joint capsule was described as a thin linear pattern [26]. Kholinne et al. [14] and Wegmann et al. [15] measured the width of the capsular attachment at the olecranon as 6.7 mm and 4.3 to 10.8 mm, respectively. Thus, the attachment pattern of the posterior elbow joint capsule at the olecranon remains inconsistent. This study showed that the width of the capsular attachment was significantly greater at the olecranon tip (approximately 9.4 mm) than at the medial and lateral edges (both approximately 3 mm), based on the precise attachment relationships among the joint capsule, small muscle bundle, and triceps brachii. Recent anatomical studies on other parts of the elbow joint have revealed that the capsular attachment was wide and varied in width depending on the location [29, 30]. The variation in the width of the capsular attachment at the olecranon described above may reflect insufficient recognition of regional differences in the joint capsule’s attachment width. In addition, prior measurements may have included the attachment of the small muscle bundle.

Our findings may be clinically relevant by providing anatomical insights into the pathophysiology of restricted elbow flexion. Restricted elbow flexion is considered to result from adhesions between the triceps brachii and the posterior elbow joint capsule [2], and elbow flexion–extension stretches or exercises targeting the triceps brachii are routinely prescribed. However, such exercises may not improve restricted elbow flexion [5, 6]. In the present study, we revealed that a small muscle bundle formed a muscular complex with the anconeus, covered the posterior elbow joint capsule and fat pad, and was separated from the triceps brachii. Moreover, we found that the thickness of the small muscle bundle changed during forearm rotation, causing compression or loosening of the posterior capsule. Tubbs et al. [16] suggested that the small muscle bundle compress the posterior capsule; however, its dynamic changes associated with elbow joint movement were unclear. Travill [19] and Pauly et al. [20] showed that the anconeus muscle was active during forearm supination. Accordingly, the complex of the small muscle bundle and anconeus, which covered the posterior capsule, may contract during forearm supination, thus compressing the posterior capsule. These findings suggest that the presence of the small muscle bundle deep to the triceps may have been neglected in previous considerations of restricted elbow flexion and may provide anatomical clues for understanding the pathophysiology underlying the persistence of restricted elbow flexion despite interventions targeting the triceps. In addition, recognition of the small muscle bundle may be useful when considering the layered anatomy of the posterior elbow during operative management of restricted elbow flexion.

This study has some limitations. First, the cadaveric specimens were obtained from elderly donors, which may not fully represent the age range of patients who develop post-traumatic or postoperative elbow stiffness. Therefore, the anatomical findings were complemented by ultrasonographic imaging in healthy young volunteers. Second, the elbow pathology presented in this study is speculative and must be verified in a clinical setting. Third, although muscle thickness changes measured using ultrasonography were interpreted as indirect indicators of contraction, no direct functional assessment was performed using electromyography or shear-wave elastography. Thus, further investigations are required to validate the functional activity of the small muscle bundle. Finally, several methodological limitations should be acknowledged. Fixation in 8% formalin and storage in 30% ethanol may affect soft tissue properties, including muscle texture, connective tissue planes, and capsular stiffness. However, previous anatomical studies regarding the elbow joint capsule used the same fixation and preservation method [29, 31, 32]. The small number of specimens in the nerve distribution group might not be sufficient to establish consistent innervation patterns. The footprint and ultrasonographic measurements were not normalized to humeral or ulnar size. Although the between-day reliability of measurements was assessed, interobserver reliability was not evaluated. Humeral rotation and probe pressure during ultrasonographic assessment were not quantitatively controlled.

## Conclusions

The small muscle bundle deep to the triceps brachii, formed a complex with the anconeus muscle, covered the posterior elbow joint capsule and fat pad, and was separated from the triceps brachii. The common tendon of the triceps brachii, the small muscle bundle, and the joint capsule had distinct attachments to the olecranon. Ultrasonographic findings indicated that the thickness of the small muscle bundle dynamically changed during forearm rotation, affecting the posterior elbow joint capsule.

## Data Availability

The datasets used and/or analyzed during the current study are available from the corresponding author on reasonable request.
